# Crystal structure and Hirshfeld analysis of poly[bis­(*N*,*O*-di­methyl­hydroxyl­ammonium) [di-μ_2_-iodido-di­iodido­plumbate(II)]]

**DOI:** 10.1107/S2056989025009661

**Published:** 2025-11-11

**Authors:** Hanna R. Petrosova, Oleksandr A. Semenikhin, Vadim A. Pavlenko, Dina D. Naumova, Mircea-Odin Apostu

**Affiliations:** a Department of Chemistry, Taras Shevchenko National University of Kyiv, Volodymyrska st. 64/13, 01601 Kyiv, Ukraine; b Department of Chemistry, Faculty of Chemistry Al. I. Cuza University of Iasi, Carol I Blvd 11, 700506 Iasi, Romania; Vienna University of Technology, Austria

**Keywords:** crystal structure, *N*,*O*-di­methyl­hydroxyl­ammonium, perovskite derivative, layered structure, Hirshfeld surface, hybrid compound, metal halides, lead(II) iodide

## Abstract

The title compound, {(C_2_H_8_NO)_2_[PbI_4_]}_*n*_, represents a layered organic–inorganic perovskite, crystallizing in space group *C*2/*c*. The asymmetric unit comprises one *N*,*O*-di­methyl­hydroxyl­ammonium cation, one Pb^2+^ cation located on a twofold rotation axis, and two iodide anions. The Pb^2+^ cation is coordinated by six iodido ligands, generating a slightly distorted octa­hedral {PbI_6_} unit. The octa­hedra are connected by corner-sharing of equatorial I^−^ ligands to form polymeric inorganic sheets extending parallel to the *ab* plane. These sheets are separated by double layers of the organic cations, producing a typical di-periodic perovskite-type arrangement with stacking of the layers along the *c* axis. Neighbouring inorganic layers are shifted relative to each other along both the *a* and *b* axes. The *N*,*O*-di­methyl­hydroxyl­ammonium cation engages in two N—H⋯I hydrogen bonds directed toward axially bound iodido ligands, which consolidates the packing of the crystal structure.

## Abstract 2

The crystal structure of (C_2_H_8_NO)_2_[PbI_4_] is layered and can be derived from the perovskite structure where the *N*,*O*-di­methyl­hydroxyl­ammonium cations are organized in layers and bound to the anionic layers through N—H⋯I inter­actions.

## 1. Chemical context

Hybrid organic–inorganic compounds with crystal structures related to perovskites have become one of the most intensively studied classes of functional materials over the past decade due to their remarkable optoelectronic properties, ease of solution processing, and compositional tunability (Kojima *et al.*, 2009 ▸; Green *et al.*, 2014 ▸; Snaith, 2013 ▸). Their potential applications span a wide range of technologies, including photovoltaics, light-emitting diodes, lasers, and photodetectors (Stranks & Snaith, 2015 ▸; Park, 2015 ▸). The structural flexibility of hybrid perovskites enables the incorporation of various organic cations, metal cations, and halide anions, giving rise to a broad spectrum of compounds with tailored physical properties (Zhao & Zhu, 2016 ▸).

A particularly important concept in this field is the periodicity of the perovskite framework. While tri-periodic perovskites, composed of extended frameworks made up from corner-sharing octa­hedral {*M*X_6_} (*M* = Pb, Sn, Ge; *X* = halide) building units, dominate the research landscape (Kucheriv *et al.*, 2025 ▸), reduced systems in periodicity, *viz*. di-periodic, perovskite-inspired mono-periodic and even zero-periodic organic–inorganic hybrids are also worth paying attention to (Mitzi, 1999 ▸; Smith *et al.*, 2014 ▸). In the most common di-periodic hybrid perovskites, the inorganic layers of corner-sharing octa­hedra are separated by organic cations, leading to natural quantum-well structures with enhanced structural stability and tunable optical and electronic properties (Ishihara, 1994 ▸; Cao *et al.*, 2015 ▸). These features make such perovskites highly promising as more stable alternatives compared to tri-periodic analogues in optoelectronic applications (Blancon *et al.*, 2018 ▸).

The *N*,*O*-di­methyl­hydroxyl­ammonium cation, (CH_3_NH_2_OCH_3_)^+^, could be intriguing for templating di-periodic structures. Studies of hydroxyl­ammonium-based salts have documented that these cations form particularly strong inter­molecular hydrogen bonds, significantly enhancing crystal packing density (Meng *et al.*, 2016 ▸). Despite these advantages, reports incorporating *N*,*O*-di­methyl­hydroxyl­ammonium in perovskites are represented by only one example (Sirenko *et al.*, 2025 ▸).
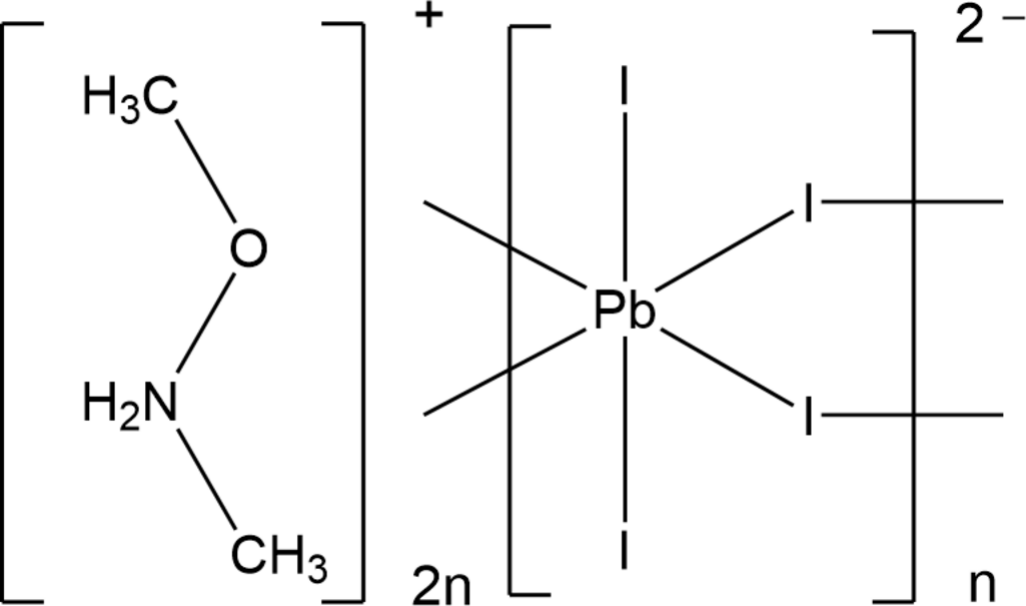


In the current work, we present the synthesis, crystal structure refinement and Hirshfeld surface analysis of (C_2_H_8_NO)_2_[PbI_4_]. Our study also highlights how the cation influences the hydrogen-bonding network, octa­hedral arrangement, and overall periodicity of the perovskite-type structure.

## 2. Structural commentary

**Figure 1:**
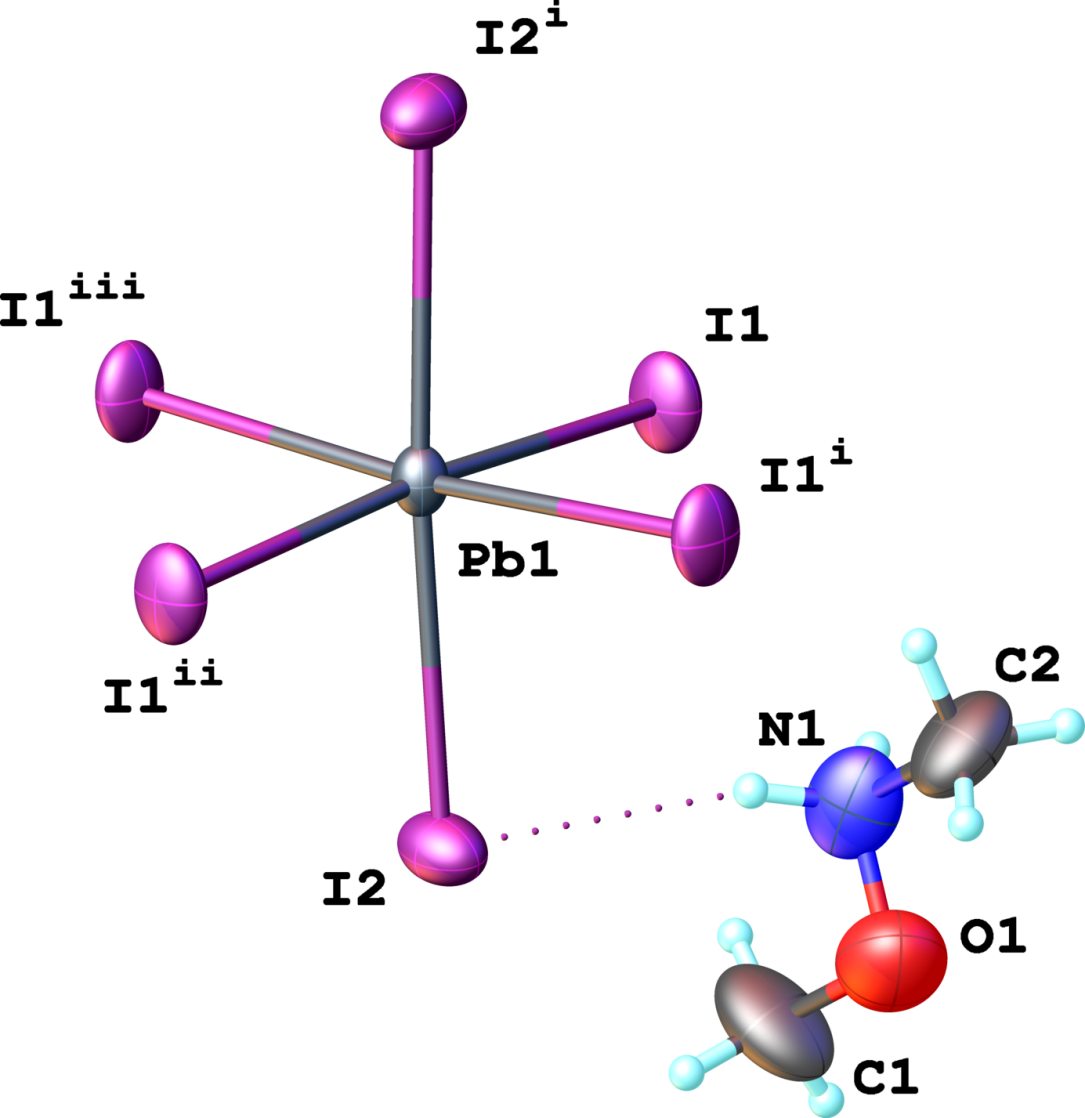
View of the basic structural units of the title compound, showing one {PbI_6_} octa­hedron and the associated organic cation with the atom-labelling scheme and displacement ellipsoids drawn at the 50% probability level; H atoms are shown as small spheres of arbitrary radius. The N—H⋯I hydrogen bond is shown as a dotted line. [Symmetry codes: (i) 1 − *x*, + *y*, 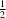 − *z*; (ii) −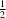 + *x*, −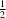 + *y*, + *z*; (iii) 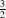 − *x*, −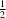 + *y*, 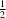 − *z*.]

In the asymmetric unit, one Pb^2+^ cation located on a twofold rotation axis, two iodide anions, and a single N,O-di­methyl­hydroxyl­ammonium cation are present (Fig. 1 ▸). The Pb^2+^ cation is sixfold coordinated by iodido ligands, giving rise to a distorted {PbI_6_} octa­hedron with Pb—I bond lengths varying between 3.1622 (16) and 3.2028 (13) Å. Bond angles confirm this distortion: *cis*-I—Pb—I bond angles are between 87.07 (5) and 95.73 (4)°, while the *trans-*I—Pb—I bond angles are 174.33 (5)° for equatorial I^−^ ligands and 176.07 (7)° for axial ones. The degree of deviation from ideal octa­hedral coordination is expressed by two parameters: Δ*d* = 1/6Σ^6^_*i*=1_(*d_i_ − d*)^2^/*d*^2^ (1) and Σ = Σ^12^_*i*=1_|90 *– α_i_*| (2), where *d_i_* is the individual bond length, *d* is average bond length and *α_i_* are twelve individual *cis*-angles in the coordination octa­hedron. For this compound, Δ*d* is determined to be 2.79 × 10^−5^, and the Σ parameter is 24.40°.

**Figure 2:**
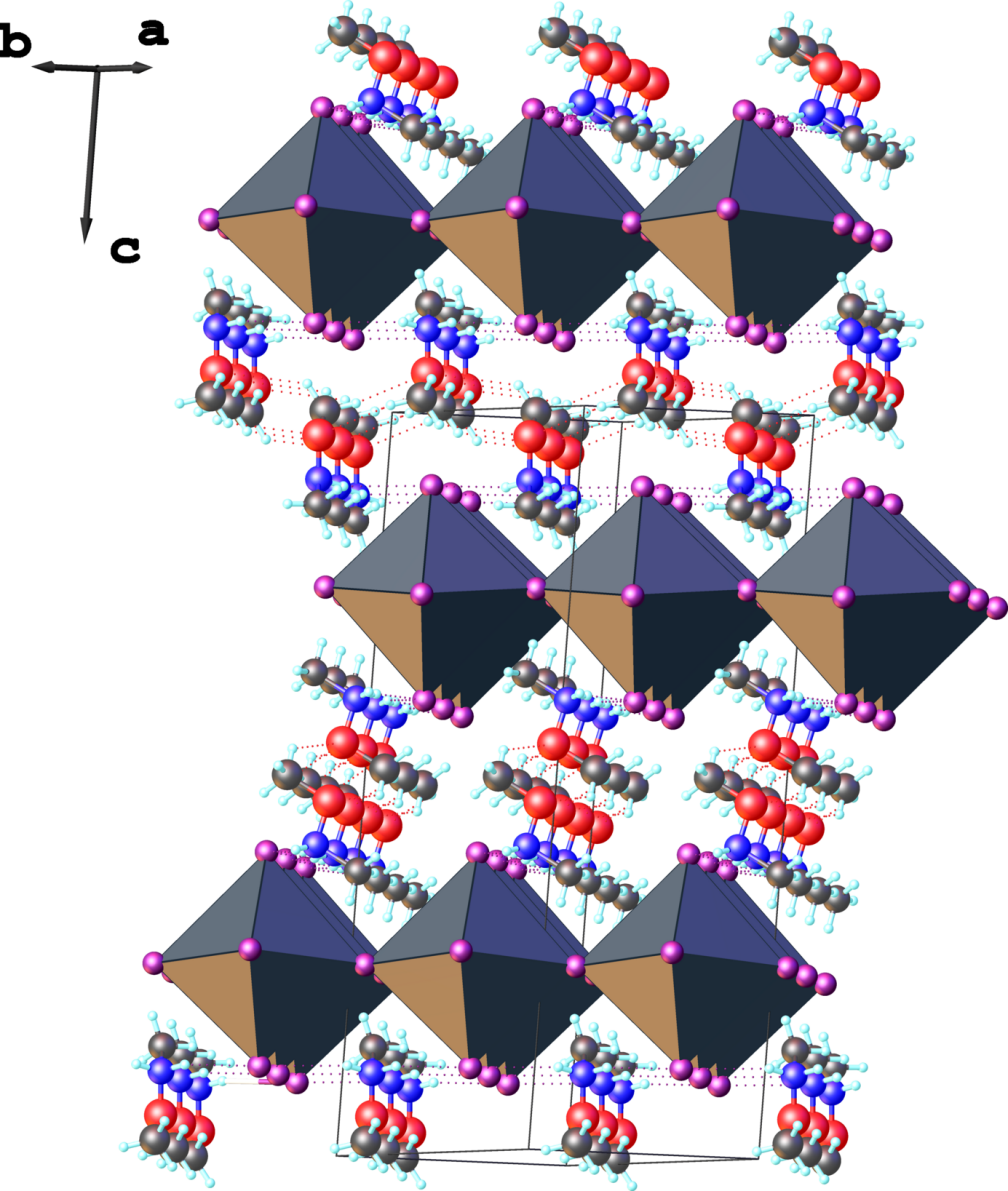
View of the crystal structure packing of (C_2_H_8_NO)_2_[PbI_4_], showing the inorganic layers and double layers of cations stacked along the *c* axis.

In the title compound, the {PbI_6_} coordination octa­hedra share equatorial corners to form polymeric inorganic layers with composition ^2^_∞_{[PbI_4/2_I_2/1_]^2–^}, which extend parallel to the *ab* plane. The organic (CH_3_NH_2_OCH_3_)^+^ cations are organized in double layers situated between the anionic sheets. The stacking of the two types of layers proceeds along the *c* axis, with the cations oriented parallel to this axis (Fig. 2 ▸).

## 3. Supra­molecular features

Inter­action between the *N*,*O*-di­methyl­hydroxyl­ammonium cations and the inorganic layers occurs through the protonated secondary amino group, which establishes N—H⋯I hydrogen bonds with the axially bound iodido ligands only (Table 1 ▸).

**Figure 3:**
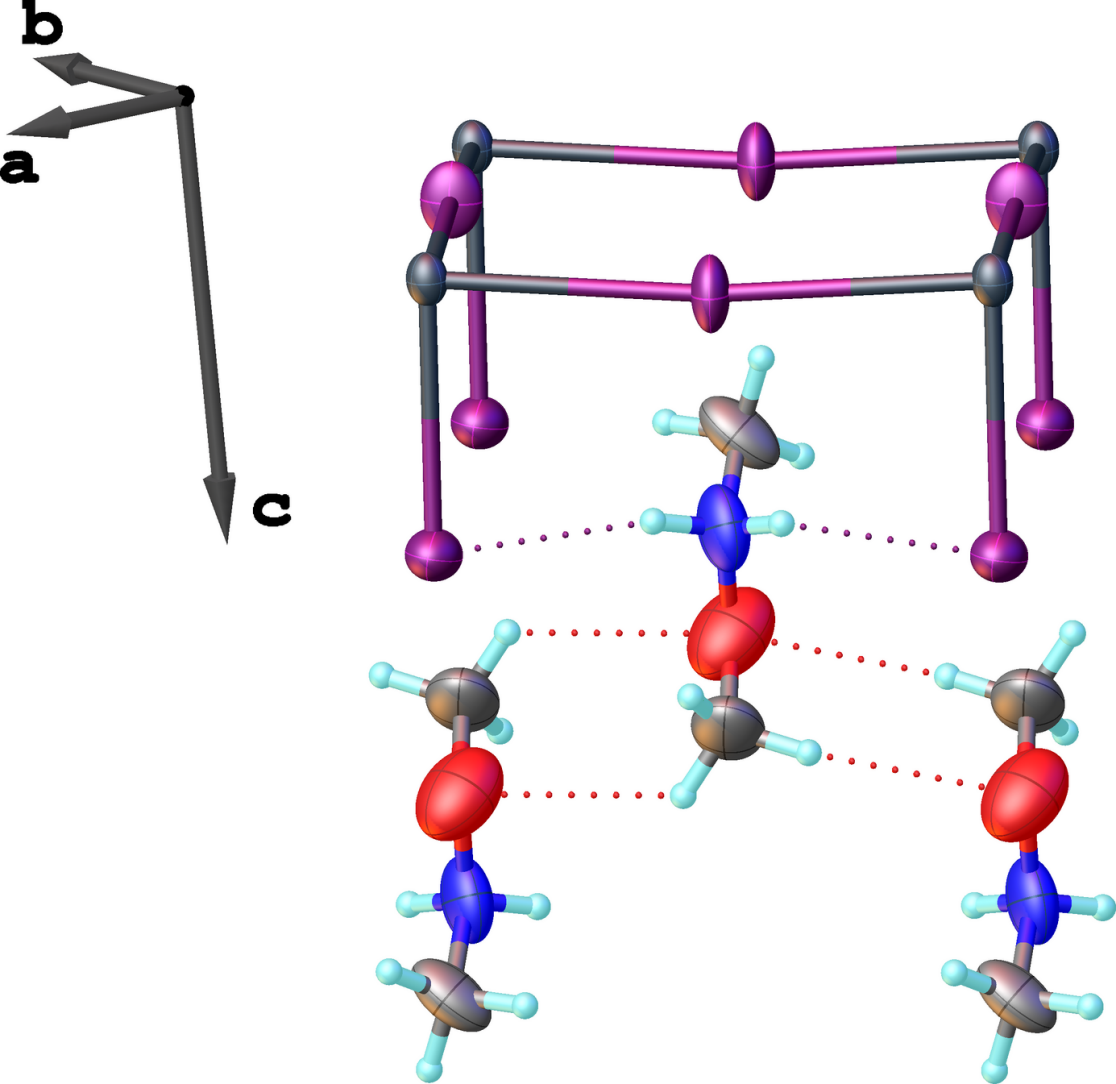
Representation of structural fragments, highlighting the hydrogen-bonding scheme in (C_2_H_8_NO)_2_[PbI_4_] (drawn as dotted lines).

The *N*,*O*-di­methyl­hydroxyl­ammonium cations are oriented perpendicularly to each other on opposite sides of the inorganic layer (Fig. 2 ▸). This perpendicular alignment reduces the distortion of the inorganic layer, unlike an arrangement where the cations would adopt parallel orientations on both sides. The observed orientation arises from the packing of adjacent inorganic sheets, which optimizes the filling of the inter­layer space occupied by the (CH_3_NH_2_OCH_3_)^+^ double layers, which additionally are linked to each other by C—H⋯O hydrogen bonds (Table 1 ▸, Fig. 3 ▸). Furthermore, the two neighbouring inorganic layers, separated by the double layers of organic cations, are shifted along both the *a* and *b* axes.

## 4. Hirshfeld surface analysis

**Figure 4:**
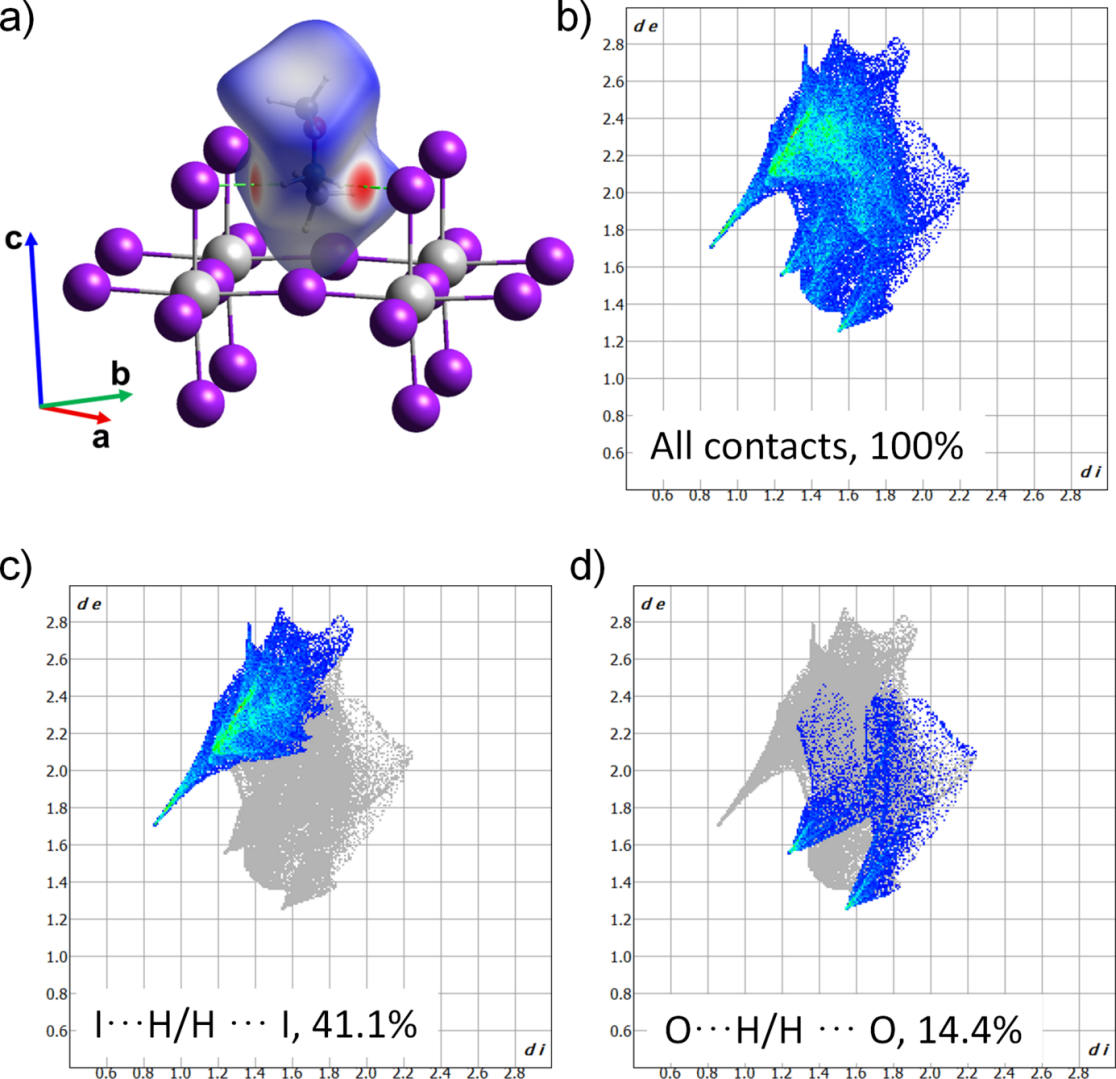
(*a*) Hirshfeld surface representation with the function *d*_norm_ plotted onto the surface for the different inter­actions. Two-dimensional fingerprint plots from the Hirshfeld surface analysis of the title compound showing: (*b*) all contacts; (*c*) I⋯H/H⋯I (41.1%); (*d*) O⋯H/H⋯O (14.4%).

To further investigate the inter­molecular contacts, a Hirshfeld surface analysis was carried out with *CrystalExplorer* (Spackman *et al.*, 2021 ▸). From this analysis, the related two-dimensional fingerprint plots were obtained. The *d*_norm_ surface displays two distinct red spots along with several white regions (Fig. 4 ▸*a).* The red–white–blue colour scheme was applied, where red regions corresponds to short inter­molecular contacts smaller than the sum of van der Waals (vdW) distances, white regions indicate close to vdW distances, and blue regions indicates longer vdW contacts. The red spots on the Hirshfeld surface are attributed to the rather strong inter­molecular N—H⋯I hydrogen bonds, whereas the white regions mainly refer to H⋯H and O⋯H/H⋯O contacts. The overall two-dimensional fingerprint plot (Fig. 4 ▸*b*) is complemented by decomposed plots for H⋯H, I⋯H/H⋯I, O⋯H/H⋯O and I⋯O/O⋯I contacts, which also show their relative contributions to the Hirshfeld surface (Fig. 4 ▸*c*,*d*). The I⋯H/H⋯I inter­actions contribute 41.1% to the crystal packing and thus define most of the relevant inter­actions in the crystal structure, while O⋯H/H⋯O inter­actions contribute 14.4% to the crystal structure. The remaining contribution originates from H⋯H contacts (44.1%), which can be found frequently in the structure, as H atoms occupy terminal positions. I⋯O/O⋯I contacts (0.4% contribution) appear to be irrelevant for the packing.

## 5. Database survey

A search of the Cambridge Structure Database (CSD version 6.00, last update August 2025; Groom *et al.*, 2016 ▸) revealed more than 2000 entries containing {PbI_6_} octa­hedra in combination with organic ammonium cations. However, only a single structure incorporating the *N*,*O*-di­methyl­hydroxyl­ammonium cation was identified, refcode MUPCIN (Sirenko *et al.*, 2025 ▸). It is isostructural with the title compound and composed of corner-sharing {SnBr_6_} octa­hedra as the inorganic component.

## 6. Synthesis and crystallization

PbI_2_ (0.21 g, 0.45 mmol) was dissolved in a mixture of 0.5 ml concentrated hydroiodic acid (57%_wt_) and 50 µl H_3_PO_2_ under heating with continuous stirring. After complete dissolution, *N*,*O*-di­methyl­hydroxyl­amine hydro­chloride (0.088 g, 0.90 mmol) was added to the mixture. Stirring was continued until a homogeneous solution was obtained. On cooling to room temperature, light-red crystals formed spontaneously. The product was collected and stored in the mother liquor until it was used for diffraction experiments.

## 7. Refinement

Crystal data, data collection and structure refinement details are summarized in Table 2 ▸. Four twin components were identified by *PLATON* (Spek, 2020 ▸) and the corresponding HKLF5 file was generated. The proportions of the twin components were determined during the refinement cycles as 0.093 (4), 0.294 (4), 0.271 (4), 0.342 (4). Hydrogen atoms in methyl groups were placed at calculated positions and refined as rotating with C—H = 0.96 Å and *U*_iso_(H) = 1.5*U*_eq_(C). Hydrogen atoms of the­amino group were placed at calculated positions and refined as riding atoms with N—H = 0.89 Å and *U*_iso_(H) = 1.2*U*_eq_(N).

## Supplementary Material

Crystal structure: contains datablock(s) I. DOI: 10.1107/S2056989025009661/wm5774sup1.cif

Structure factors: contains datablock(s) I. DOI: 10.1107/S2056989025009661/wm5774Isup2.hkl

CCDC reference: 2499482

Additional supporting information:  crystallographic information; 3D view; checkCIF report

## Figures and Tables

**Table 1:** Hydrogen-bond geometry (Å, °)

*D*—H⋯*A*	*D*—H	H⋯*A*	*D*⋯*A*	*D*—H⋯*A*
N1—H1*A*⋯I2	0.89	2.81	3.674 (18)	162
N1—H1*B*⋯I2^i^	0.89	2.69	3.560 (18)	167
C1—H1*D*⋯O1^ii^	0.96	2.89	3.64 (3)	136
C1—H1*E*⋯O1^iii^	0.96	2.92	3.59 (4)	128

Symmetry codes: (i) 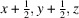; (ii) 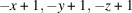; (iii) 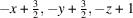.

**Table 2:** Experimental details

Crystal data	
Chemical formula	(C_2_H_8_NO)_2_[PbI_4_]
*M*_r_	838.98
Crystal system, space group	Monoclinic, *C*2/*c*
Temperature (K)	293
*a*, *b*, *c* (Å)	8.9536 (8), 8.9538 (6), 22.300 (2)
β (°)	95.751 (8)
*V* (Å^3^)	1778.8 (3)
*Z*	4
Radiation type	Mo *K*α
μ (mm^−1^)	16.41
Crystal size (mm)	0.05 × 0.05 × 0.01

Data collection	
Diffractometer	Xcalibur, Eos
Absorption correction	Multi-scan (*CrysAlis PRO*; Rigaku OD, 2022 ▸)
*T*_min_, *T*_max_	0.377, 1.000
No. of measured, independent and observed [*I* > 2σ(*I*)] reflections	2043, 2043, 1393
*R*_int_	0.065
(sin θ/λ)_max_ (Å^−1^)	0.686

Refinement	
*R*[*F*^2^ > 2σ(*F*^2^)], *wR*(*F*^2^), *S*	0.060, 0.144, 1.05
No. of reflections	2043
No. of parameters	65
No. of restraints	3
H-atom treatment	H-atom parameters constrained
Δρ_max_, Δρ_min_ (e Å^−3^)	1.81, −1.91

Computer programs: *CrysAlis PRO* (Rigaku OD, 2022 ▸), *SHELXT* (Sheldrick, 2015*a* ▸), *SHELXL* (Sheldrick, 2015*b* ▸), *OLEX2* (Dolomanov *et al.*, 2009 ▸) and *publCIF* (Westrip, 2010 ▸).

## supplementary crystallographic information

### Poly[bis(N,O-dimethylhydroxylammonium) [di-µ2-iodido-diiodidoplumbate(II)]] Crystal data

**Table:**

(C_2_H_8_NO)_2_[PbI_4_]	*F*(000) = 1456
*M**_r_* = 838.98	*D*_x_ = 3.133 Mg m^−^^3^
Monoclinic, *C*2/*c*	Mo *K*α radiation, λ = 0.71073 Å
*a* = 8.9536 (8) Å	Cell parameters from 1334 reflections
*b* = 8.9538 (6) Å	θ = 3.2–28.4°
*c* = 22.300 (2) Å	µ = 16.41 mm^−^^1^
β = 95.751 (8)°	*T* = 293 K
*V* = 1778.8 (3) Å^3^	Plate, clear light red
*Z* = 4	0.05 × 0.05 × 0.01 mm

### Poly[bis(N,O-dimethylhydroxylammonium) [di-µ2-iodido-diiodidoplumbate(II)]] Data collection

**Table:**

Xcalibur, Eos diffractometer	2043 independent reflections
Radiation source: fine-focus sealed X-ray tube, Enhance (Mo) X-ray Source	1393 reflections with *I* > 2σ(*I*)
Graphite monochromator	*R*_int_ = 0.065
Detector resolution: 16.1593 pixels mm^-1^	θ_max_ = 29.2°, θ_min_ = 1.8°
ω scans	*h* = −12→12
Absorption correction: multi-scan (CrysAlisPro; Rigaku OD, 2022)	*k* = −12→12
*T*_min_ = 0.377, *T*_max_ = 1.000	*l* = −30→30
2043 measured reflections	

### Poly[bis(N,O-dimethylhydroxylammonium) [di-µ2-iodido-diiodidoplumbate(II)]] Refinement

**Table:**

Refinement on *F*^2^	Primary atom site location: dual
Least-squares matrix: full	Hydrogen site location: inferred from neighbouring sites
*R*[*F*^2^ > 2σ(*F*^2^)] = 0.060	H-atom parameters constrained
*wR*(*F*^2^) = 0.144	*w* = 1/[σ^2^(*F*_o_^2^) + (0.046*P*)^2^ + 3.2533*P*] where *P* = (*F*_o_^2^ + 2*F*_c_^2^)/3
*S* = 1.05	(Δ/σ)_max_ < 0.001
2043 reflections	Δρ_max_ = 1.81 e Å^−^^3^
65 parameters	Δρ_min_ = −1.91 e Å^−^^3^
3 restraints	

### Poly[bis(N,O-dimethylhydroxylammonium) [di-µ2-iodido-diiodidoplumbate(II)]] Special details

**Table:**

Geometry. All esds (except the esd in the dihedral angle between two l.s. planes) are estimated using the full covariance matrix. The cell esds are taken into account individually in the estimation of esds in distances, angles and torsion angles; correlations between esds in cell parameters are only used when they are defined by crystal symmetry. An approximate (isotropic) treatment of cell esds is used for estimating esds involving l.s. planes.
Refinement. Refined as a 4-component twin.

### Poly[bis(N,O-dimethylhydroxylammonium) [di-µ2-iodido-diiodidoplumbate(II)]] Fractional atomic coordinates and isotropic or equivalent isotropic displacement parameters (Å^2^)

**Table:**

	*x*	*y*	*z*	*U*_iso_*/*U*_eq_	
Pb1	0.500000	0.18968 (14)	0.250000	0.0291 (3)	
I1	0.75260 (19)	0.43769 (17)	0.25702 (6)	0.0514 (4)	
I2	0.5225 (2)	0.20194 (18)	0.39416 (6)	0.0529 (4)	
O1	0.622 (3)	0.624 (2)	0.4578 (8)	0.103 (8)	
N1	0.641 (2)	0.595 (2)	0.3990 (8)	0.069 (6)	
H1A	0.626129	0.498099	0.390777	0.083*	
H1B	0.732660	0.619761	0.390781	0.083*	
C2	0.524 (3)	0.689 (3)	0.3634 (10)	0.075 (8)	
H2A	0.508029	0.651308	0.322888	0.113*	
H2B	0.558651	0.790214	0.362640	0.113*	
H2C	0.432024	0.684702	0.381801	0.113*	
C1	0.726 (3)	0.520 (3)	0.4929 (10)	0.090 (10)	
H1C	0.804448	0.491127	0.469112	0.135*	
H1D	0.671575	0.433226	0.503564	0.135*	
H1E	0.768524	0.569215	0.528928	0.135*	

### Poly[bis(N,O-dimethylhydroxylammonium) [di-µ2-iodido-diiodidoplumbate(II)]] Atomic displacement parameters (Å^2^)

**Table:**

	*U*^11^	*U*^22^	*U*^33^	*U*^12^	*U*^13^	*U*^23^
Pb1	0.0223 (12)	0.0246 (13)	0.0405 (5)	0.000	0.0032 (7)	0.000
I1	0.0349 (15)	0.0373 (17)	0.0827 (10)	−0.0157 (5)	0.0090 (11)	−0.0014 (11)
I2	0.0619 (13)	0.0579 (12)	0.0377 (7)	−0.0162 (8)	−0.0005 (9)	0.0023 (8)
O1	0.13 (2)	0.092 (19)	0.101 (16)	−0.005 (14)	0.053 (16)	−0.003 (16)
N1	0.038 (14)	0.070 (17)	0.100 (16)	−0.014 (12)	0.010 (12)	−0.013 (15)
C2	0.07 (3)	0.08 (3)	0.068 (16)	0.008 (15)	−0.017 (18)	−0.034 (16)
C1	0.08 (3)	0.10 (3)	0.08 (2)	−0.018 (17)	−0.034 (19)	0.04 (2)

### Poly[bis(N,O-dimethylhydroxylammonium) [di-µ2-iodido-diiodidoplumbate(II)]] Geometric parameters (Å, º)

**Table:**

Pb1—I1	3.1622 (16)	N1—H1B	0.8900
Pb1—I1^i^	3.1623 (15)	N1—C2	1.504 (10)
Pb1—I1^ii^	3.1767 (15)	C2—H2A	0.9600
Pb1—I1^iii^	3.1767 (16)	C2—H2B	0.9600
Pb1—I2^i^	3.2028 (13)	C2—H2C	0.9600
Pb1—I2	3.2028 (13)	C1—H1C	0.9600
O1—N1	1.363 (9)	C1—H1D	0.9600
O1—C1	1.481 (10)	C1—H1E	0.9600
N1—H1A	0.8900		
			
I1—Pb1—I1^i^	90.79 (8)	O1—N1—H1B	110.8
I1^i^—Pb1—I1^iii^	90.138 (7)	O1—N1—C2	104.7 (17)
I1—Pb1—I1^iii^	174.33 (5)	H1A—N1—H1B	108.9
I1^i^—Pb1—I1^ii^	174.33 (5)	C2—N1—H1A	110.8
I1—Pb1—I1^ii^	90.138 (6)	C2—N1—H1B	110.8
I1^iii^—Pb1—I1^ii^	89.49 (8)	N1—C2—H2A	109.5
I1^ii^—Pb1—I2	95.73 (4)	N1—C2—H2B	109.5
I1^iii^—Pb1—I2	87.07 (5)	N1—C2—H2C	109.5
I1^iii^—Pb1—I2^i^	95.73 (4)	H2A—C2—H2B	109.5
I1^i^—Pb1—I2^i^	87.34 (4)	H2A—C2—H2C	109.5
I1—Pb1—I2	87.34 (4)	H2B—C2—H2C	109.5
I1^i^—Pb1—I2	89.90 (4)	O1—C1—H1C	109.5
I1^ii^—Pb1—I2^i^	87.07 (5)	O1—C1—H1D	109.5
I1—Pb1—I2^i^	89.90 (4)	O1—C1—H1E	109.5
I2—Pb1—I2^i^	176.07 (7)	H1C—C1—H1D	109.5
Pb1—I1—Pb1^iv^	174.33 (5)	H1C—C1—H1E	109.5
N1—O1—C1	104.9 (17)	H1D—C1—H1E	109.5
O1—N1—H1A	110.8		
			
C1—O1—N1—C2	174 (2)		

Symmetry codes: (i) −*x*+1, *y*, −*z*+1/2; (ii) −*x*+3/2, *y*−1/2, −*z*+1/2; (iii) *x*−1/2, *y*−1/2, *z*; (iv) *x*+1/2, *y*+1/2, *z*.

### Poly[bis(N,O-dimethylhydroxylammonium) [di-µ2-iodido-diiodidoplumbate(II)]] Hydrogen-bond geometry (Å, º)

**Table:**

*D*—H···*A*	*D*—H	H···*A*	*D*···*A*	*D*—H···*A*
N1—H1*A*···I2	0.89	2.81	3.674 (18)	162
N1—H1*B*···I2^iv^	0.89	2.69	3.560 (18)	167
C1—H1*D*···O1^v^	0.96	2.89	3.64 (3)	136
C1—H1*E*···O1^vi^	0.96	2.92	3.59 (4)	128

Symmetry codes: (iv) *x*+1/2, *y*+1/2, *z*; (v) −*x*+1, −*y*+1, −*z*+1; (vi) −*x*+3/2, −*y*+3/2, −*z*+1.
